# Effects of exogenous organic matter addition on agricultural soil microbial communities and relevant enzyme activities in southern China

**DOI:** 10.1038/s41598-023-33498-0

**Published:** 2023-05-17

**Authors:** Xing Liu, Qi Chen, Huicheng Zhang, Jiaen Zhang, Yuting Chen, Fucheng Yao, Yingtong Chen

**Affiliations:** 1 Guangdong Engineering Technology Research Center of Modern Eco-agriculture and Circular Agriculture, South China Agricultural University, Guangzhou, 510642 China; 2 Guangdong Laboratory for Lingnan Modern Agriculture, Guangdong Provincial Key Laboratory of Eco-circular Agriculture, South China Agricultural University, Guangzhou, 510642 China; 3 Department of Ecology, College of Natural Resources and Environment, South China Agricultural University, Guangzhou, 510642 China; 4 Key Laboratory of Agro-Environment in the Tropics, Ministry of Agriculture and Rural Affairs, South China Agricultural University, Guangzhou, 510642 China

**Keywords:** Agroecology, Environmental impact

## Abstract

Soil microbial community composition plays a key role in the decomposition of organic matter, while the quality of exogenous organic matter (EOM: rice straw, roots and pig manure) can influence soil chemical and biological properties. However, the evidences of the effect of combination of crop residues and pig manure on the changes in soil microbial community and enzymes activities are scarce. A greenhouse pot experiment was conducted to investigate the potential effect of EOM by analyzing soil properties, enzyme activities and microbial communities. The experiment consisted of eight treatments: CK (control), S (1% (w/w) rice straw), R (1% (w/w) rice root), SR (1% (w/w) rice straw + 1% (w/w) rice root), and added 1% (w/w) pig manure to CK, S, R and SR, respectively. Results showed that the straw treatment significantly increased the microbial biomass (carbon and nitrogen) and total carbon and nitrogen contents, cellulase and β-1,4-glucosidase activities, bacteria (i.e., gram-positive bacteria and gram-negative bacteria) PLFAs contents relative to CK regardless of whether pig manure was added. Moreover, the interaction between crop residues (e.g., straw and roots) and pig manure significantly influenced the contents of microbial biomass nitrogen and microbial biomass phosphorus, and the ratio of gram-positive bacteria to gram-negative bacteria. Redundance analysis confirmed that pH, nitrate nitrogen, ammonium nitrogen and dissolve organic carbon contents were significantly associated with soil microbial community under crop residues without pig manure addition. Furthermore, the experiment results showed that pig manure application not only provided more abundant nutrients (C, N and P) but also induced higher microbial and enzymatic activity compared with no pig manure addition. Our findings suggest that the combination of above-ground straw and pig manure is a better option for improving the functions of soil ecosystem.

## Introduction

Land use changes such as converting conventional tillage to no-tillage or minimal tillage, and paddy fields to upland field cultivation can cause changes in the soil properties and function^1,2^. Doubts remain uncertain how crop residues (i.e., root and straw) returning affect the availability of soil nutrients and the stability of soil carbon (C) supply under the land use change, and what difference in the contribution to soil fertility and soil organism community between above-ground straw and below-ground roots. The residues (i.e., root and straw) of previous crops, as an important and abundant biomass resource, not only directly increases the C input in agro-ecosystems, but also affects soil physical, chemical and biological properties and crop growth. However, the adverse effects of crop residues return on the quality of cultivated land, and the emergence, growth and development of crops have also been reported^3^. Thus, it is necessary to optimize fertilization methods, such as combining crop residues and animal wastes, to compensate for the deficiency of nitrogen in crop residues and to eliminate the competition with crops for nutrients during the decomposition of the residues.

The crop residues are increasing every year and annually around 360 MT in the world, of which the production of rice straw is about 234 MT in China^3^. Specifically, rice straw is rich in nutrients that can be degraded by microorganisms, and can contribute with 2.1 ~ 2.2 t C ha^−1^, 31 ~ 42 kg N ha^−1^, 8 kg P ha^−1^ and 34 ~ 61 kg K ha^−1^ per crop cycle by returning to the field^4,5^. Although the contribution of above-ground rice straw to SOC sequestration is significant, rice roots have been reported to contribute to SOC one point five to three fold than that of rice straw^6,7^. Generally, the decomposition rate of rice roots is lower than straw, this may be explained by the higher content of recalcitrant components such as lignin and lower content of decomposable components such as non-structural carbohydrates^6,8^. Conversely, the improper disposal, utilization, and management practices of crop residues could lead to environmental problem such as greenhouse gases emissions, air pollution and public health issues. In addition, the intensification of livestock has resulted in a large increase in the production of animals waste, but animals manure (e.g., pig manure) contains a large amount of organic matter, nitrogen (N) and phosphorus (P)^9^, which may largely compensate for the imbalances of soil nutrients caused by high intensity agricultural production^10,11^. It is well-known that organic fertilizer such as pig manure is considered worldwide as a promising and inexpensive source of nutrients to meet crop nutrient demand, maintain soil fertility and achieve high crop yield^10^. Thus, the rational use of crop residues and animal wastes not only reduces nutrients loss but also alleviates various environmental problems (e.g., greenhouse gases emissions, water and air pollution).

Organic matter decomposition and soil nutrient turnover cannot be achieved without the participation of extracellular enzymes, while the activity of extracellular enzymes can serve to evaluate soil health status^12,13^. Previous research work has reported that soil extracellular enzymes are synthetized and secreted by soil microorganisms, and are direct drivers of organic matter decomposition^13^. Soil enzymes may be a good indicator of soil biological changes as they respond rapidly to variables in soil fertility^14^. Moreover, soil enzyme activity may be a rate-limiting step in mineralization process and has a rapid response to soil C and nutrient availability with fertilizer^15^. In the cycling of nutrients, some soil enzymes participate in the elemental biochemical process, such as cellulase and β-1,4-glucosidase (BG) play an important role in degrading cellulose, producing glucose and releasing available nutrients, while *N*-acetyl-β-Dglucosaminidase (NAG) and Urease can represent soil N turnover, and acid phosphomonoesterase (AcP) is involved in organic phosphorus hydrolysis^16–19^. These enzymes are sensitive indicators for the variables of soil properties and are useful for the sustainable management of soil quality^14^. Therefore, soil extracellular enzymes participate in the decomposition of organic matter and modulate the rate-limiting steps of N and P mineralization in soils.

Soil microorganism is an important regulator of organic materials decomposition, while the addition of exogenous organic matter (EOM) affects microbial community composition^12,20,21^. Moreover, soil microorganism also plays an important role in the ecosystem processes, such as soil structural formtion and nutrients turnover, thereby maintaining soil agroecosystem functioning and sustainability^12,22^. The variation in critical species engaged in nutrient cycling can also impact soil fertility, for example, bacteria dominate in the initial stages of degrading organic matter, while fungi and actinomycetes can degrade the recalcitrant lignocellulose components and dominate in the later stage^23,24^. Several studies have reported that EOM (e.g., pig manure and crop residues) addition significantly affected microbial community composition and diversity^9,25,26^. Additionally, changes in microbial community structure and activity are important for SOM pools since the secretions (e.g., soil enzyme) from different microbial groups are involved in the dynamics of C in soil^21^. In general, crop residue inputs to soil are considered to provide substrates that are more readily utilizable than native SOM for microorganisms^12,20^, this might be related to a large proportion of chemical complex and recalcitrant to decomposition in native SOM^27^. As we all know, pig manure could strongly influence microbial communities by shifting oligotrophic organisms (common in exclusively mineral fertilizer or soils without fertilizer) toward microbes decomposing complex organic compounds^28^. This occurs because pig manure is rich in organic matter and nutrients, which are conducive to the metabolism and growth of microorganisms^9^. Therefore, it is necessary to further explore the effects of crop residues and their combined addition with pig manure on soil microbial community composition and diversity.

Returning crop residues (i.e., rice straw and root) and pig manure can provide nutrients and organic matter to meet the growth and metabolic requirement of crops, and maintain soil fertility. However, whether pig manure addition can wake soil enzyme activity and microbial community composition response to crop residues remains unclear. Therefore, it is crucial to investigate the potential impact of EOMs (i.e., pig manure, above-ground straw and below-ground root) on microbial community composition, as well as the involved enzymatic processes that control the availability of most limiting nutrients for microbial metabolic requirements. In this study, EOMs were collected to devise a greenhouse pot experiment, we hypothesized that the combination addition of crop residues (i.e., rice straw and root) and pig manure would change microbial community composition and enzyme activity. Specifically, the objectives of this study were (1) to determine the effect of crop residues (i.e., above-ground straw and below-ground root and their combination with pig manure on soil properties, enzyme activities and soil microbial community, (2) to estimate the relationships between soil microbial community and soil properties and enzyme activities, (3) to explore the benefits of above-ground straw and below-ground root in combination with pig manure to optimize its agronomic performance.

## Materials and methods

### Preparation of experimental soil

The soil for this study  came from the Zengcheng Teaching and Research Farm (23°14′22″N, 113° 37′57″E), South China Agriculture University, Guangzhou, China. The climatic condition of the sampling site belongs to a typical subtropical monsoon climate and the soil is classified as sandy clay, consisting of sand (57.56%), silt (6.13%) and clay (36.31%), as well as the main soil properties are shown in Table 1.

**Table 1:** Chemical properties of the tested soil, exogenous organic matter and pig manure.

Material	Total carbon (g·kg^−1^)	Total nitrogen (g·kg^−1^)	Total phosphorus (g·kg^−1^)	pH	Total carbon to nitrogen ratio (C/N)
Soil	16.78	1.92	0.65	5.69	8.74
Rice straw	52.28	0.92	2.62	/	56.83
Rice root	12.04	0.60	2.41	/	20.07
Pig manure	49.79	1.10	15.19	/	45.26

On 15 November, 2021, the soil was collected in the surface soil (0 ~ 20 cm) from paddy fields after rice harvest. Soil samples were transported to the laboratory, and the visible stones and plant roots were removed. Meanwhile, we also collected the post-harvest straw and roots from the same plot, and transported to the laboratory for cleaning. The collected samples including rice straw, roots and animal manure (pig manure) were oven-dried at 105℃, and then crushed, homogenized, ground and stored in a fine powder, and through a 2-mm sieve before the pot experiment. The properties of straw, roots and pig manure are shown in Table 1.

### Experiment design

The greenhouse pot experiment started on December 8, 2021 and conducted in a greenhouse at South China Agriculture University, Guangzhou, China. Each plastic pot (height = 200 mm, a bottom diameter = 160 mm and a top diameter = 213 mm) contains 3.5 kg of naturally air-dried soil through a 2-mm sieve, and a filter paper beneath the bottom to prevent the soil loss. Subsequently, we set up eight treatments with six replicates: unamended control soil (denoted by CK), soil + 1% (w/w) rice straw (denoted by S), soil + 1% (w/w) rice root (denoted by R), soil + 1% (w/w) rice straw + 1% (w/w) rice roots (denoted by SR), and added 1% (w/w) pig manure to CK, S, R and SR, respectively. Maize was planted as the test crop in each plastic pot, and the average greenhouse temperature was maintained at 25 °C, the growing process of maize lasted 136 days. Watering was done little and frequently in the beginning to ensure smooth seed germination. During growth, a moderate amount of deionized water was sprayed on the surface of each pot weekly so that the soil moisture content was sufficient for maize growth and metabolism.

### Soil sampling

On April 22, 2022, soil samples were destructively collected at the end of the maturation period by drilling three cores (3 cm in diameter) from each plastic pot, and then six cores from two replicates were mixed to form a composite one. Each soil sample composite was homogenized and passed through a 2-mm sieve, and the sieved soil is divided into three parts. A portion of the soil sample was stored at 4 °C for subsequent content analysis of soil microbial biomass carbon (MBC), microbial biomass nitrogen (MBN), microbial biomass phosphorus (MBP), dissolving organic carbon (DOC), ammonium nitrogen (NH_4_^+^−N) and nitrate nitrogen (NO_3_^−^–N). A portion of soil sample was air-dried for determining soil pH, and contents of soil total carbon (TC), total nitrogen (TN), total phosphorus (TP) and available phosphorus (AP), and enzyme activities. The last portion of soil sample was freeze-dried to extract the phospholipid fatty acids (PLFA).

### Soil properties and microbial biomass analysis

Soil pH was determined using the digital pH meter (Seven2Go, Mettler-Toledo Instruments (Shanghai) Co., Ltd, Shanghai, China) in a 1:2.5soil /water suspension after shaking at 250 rpm for 5 min. TC and TN contents were determined using a Vario micro-Cube elemental analyzer (Analyzer Vario MICRO cube, Elementar, Germany). Soil NO_3_^−^–N and NH_4_^+^−N were extracted by 2 M KCl and their contents were determined using a colorimetric method by an AutoAnalyser III continuous Flow Analyzer (Bran + Luebbe, AA3 AutoAnalyzer, German). MBC and MBN contents were determined using the chloroform fumigation extraction method and the extracted liquid was determined by a TOC analyzer (Multi C/N 3000, Analytik Jena, Germany)^29^. The TP content was determined by the antimony molybdenum anti-colorimetric method after perchloric acid digestion, MBP was extracted with 0.5 M NaHCO_3_ and its content was measured using the chloroform fumigation extraction method, AP was also extracted with 0.5 M NaHCO_3_, all of the production were detected by antimony molybdenum anti-colorimetric method described by Bray and Kurtz^30^. DOC was extracted with 0.5 M K_2_SO_4_ after shaking at 200 rpm for 1 h, then the filtrates were used to determine the DOC content in the Vario TOC element analyzer (Elementar, Hanau, Germany).

### Soil enzyme activities analysis

Soil enzyme (cellulase, β-1,4-glucosidase [BG], Urease, N-acetyl-β-D-glucosaminidase [NAG] and acid phosphomonoesterase [AcP]) activities were determined by using an enzyme activity detection kit (Nanjing Mol Farming Biotechnology Co., Ltd.). The substrates for enzymes were as follows: Cellulase, BG, Urease, NAG and AcP were assayed with the 3.5-dinitrosalicylic acid (DNS)^31^, ρ-nitrophenyl-β-D-glucopyranoside^32^, urea^33^, ρ-nitrophenyl-*N*-acetyl-β-d-glucosaminide^34^, and 4-methylumbelliferyl-phosphate^33^, respectively. For detailed determination of the above five soil enzymes refers to the enzyme activity detection kit procedures.

### Analysis of PLFA

Microbial community structure was assessed by using phospholipid fatty acids (PLFA) analysis^21,35^. Briefly, the PLFAs were extracted from 8 g of freeze-dried soil with chloroform–methanol–citrate buffer mixture (1:2:0.8), followed by elution with chloroform, acetone and methanol. After the organic phase separation, purification, and methyl esterification, the PLFAs were analyzed using an Agilent 7890A GC (Version 6.2, MIDI Inc., Newark, Delaware, USA). MIDI software (“Sherlock Microbial Identification System”) was used to identify the individual PLFAs peaks and quantify the peak areas with reference to the internal standard peak (19:0) of known concentration. The biomarkers mainly included gram-positive bacteria (a11:0, a13:0, i14:0, i15:0, a15:0, i15:1 ω6c, i16:0, i17:0, a17:0, i17:1 ω9c and i18:0), the gram-negative bacteria (16:1 ω9c, 16:1 ω7c, 17:1 ω8c, 18:1 ω7c, 18:1 ω5c, cy17:0 ω7c and cy19:0 ω7c), saprophytic fungi (18:1 ω9c and 18:2 ω6c), arbuscular mycorrhizal fungi (16:1 ω5c) and actinomycetes (16:0 10-methyl, 17:0 10-methyl, 17:1 ω7c 10-methyl, 18:0 10-methyl and 20:0 10-methyl). The sum of gram-positive bacteria (G+), gram-negative bacteria (G−) and non-specific bacteria (14:0, 15:0, 15:0 DMA, 16:0, 16:3 ω6c and 18:0) was used as total bacteria. All the above PLFAs were considered to be representative of the total PLFAs of the soil microbial community^21^. The PLFAs abundance was expressed in nmol g^−1^ dry soil.

### Statistical analysis

All values were based on the weight of oven-dried soil (105℃) and the data were averaged from three replicates with standard deviation. The effect of EOM on soil properties, enzyme activities, microbial biomass and community was investigated by two-way analysis of variance (Two-way ANOVA) using the “car” package in R software. When the interactions between crop residues and pig manure were significant, they were further analyzed by using a one-way ANOVA (Duncan or multiple comparison) and independent-samples t-test, respectively. The only main effect was compared when the interaction was not significant. The significance level was set at *p* < 0.05.

Pearson correlation analysis was performed to analyze the relationship between soil microbial community and enzyme activities, and the “corrplot” package in R was used to analyze soil properties, microbial biomass, enzyme activity and microbial community. Redundancy analysis (RDA) was conducted by using the “vegan” package in R software to explore the relationships between soil microbial community, soil chemical and biological properties.

## Results

### Soil properties and microbial biomass under the EOM additions

**Figure 1:**
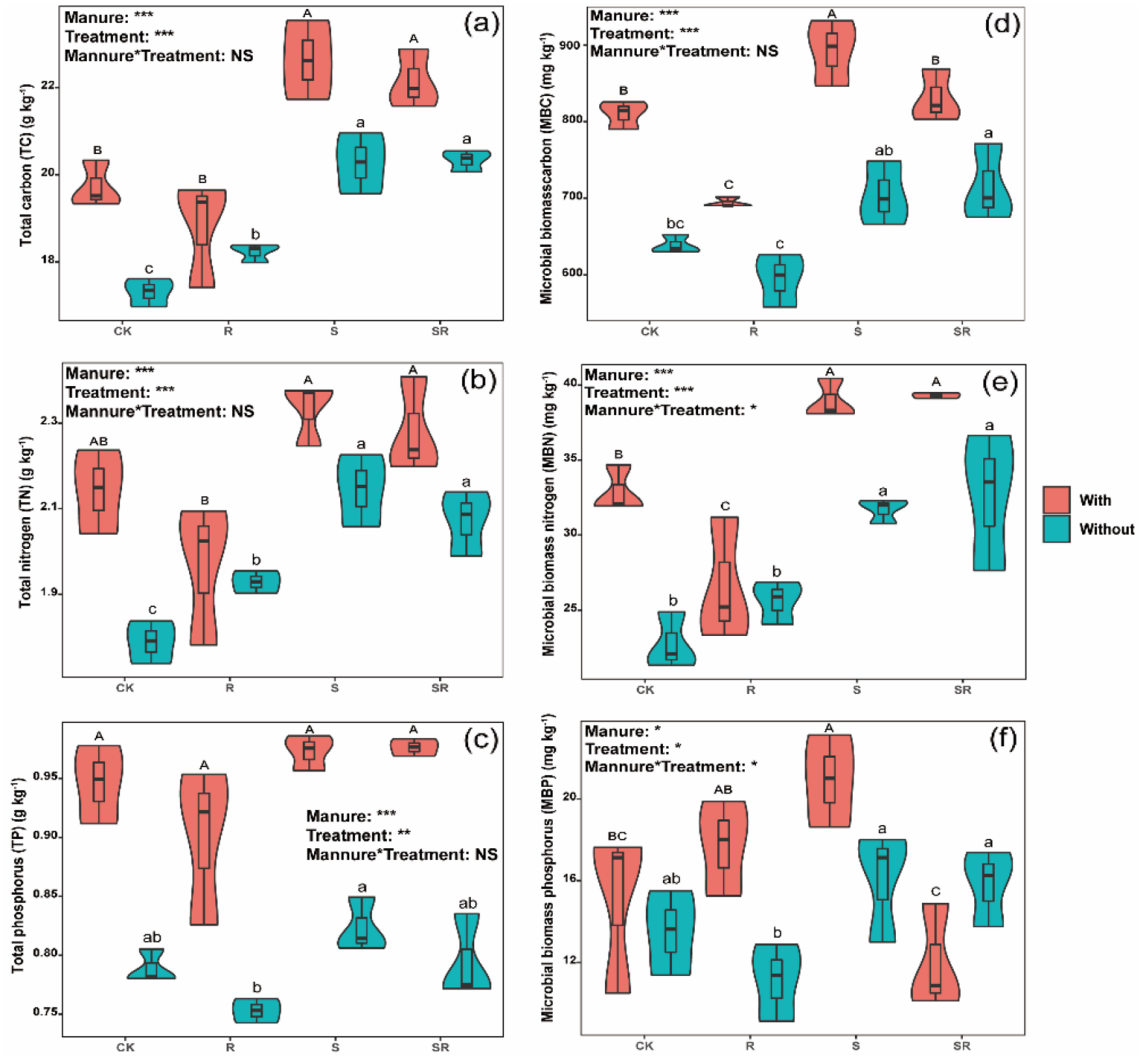
Effect of exogenous organic matter (EOM) on the contents of soil total nutrients (carbon, nitrogen and phosphorus) and microbial biomass (carbon, nitrogen and phosphorus), and the plots were labeled without pig manure (without), with pig manure (with). The data were expressed by mean ± standard error (n = 3). Pig manure × Treatment: interaction between crop residues without and with pig manure. The different lowercase and uppercase letters denote the significant difference among different treatments without and with pig manure addition at 0.05 level, respectively. The two-way ANOVA results were indicated by NS non-significant, *p < 0.05, **p < 0.01, ***p < 0.001.

**Figure 2:**
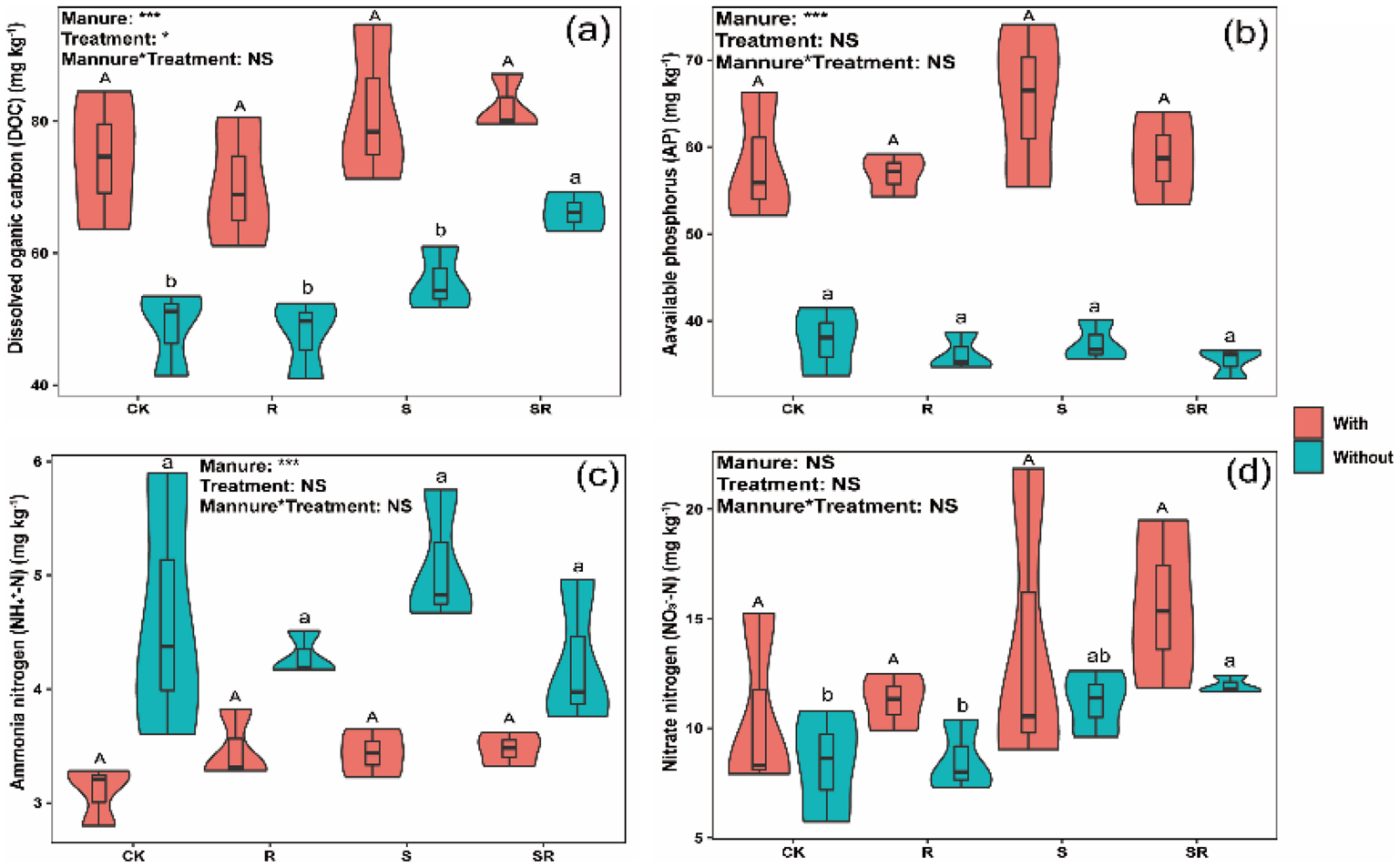
Effect of EOM on contents of soil available nutrients (DOC, AP, NH_4_^+^–N and NO_3_^−^–N), and the plots were labeled without pig manure (without), with pig manure (with). The data were expressed by mean ± standard error (n = 3). Pig manure × Treatment: interaction between crop residues without and with pig manure. The different lowercase and uppercase letters denote the significant difference among different treatments without and with pig manure addition at 0.05 level, respectively. The two-way ANOVA results were indicated by NS non-significant, *p < 0.05, **p < 0.01, ***p < 0.001.

**Figure 3:**
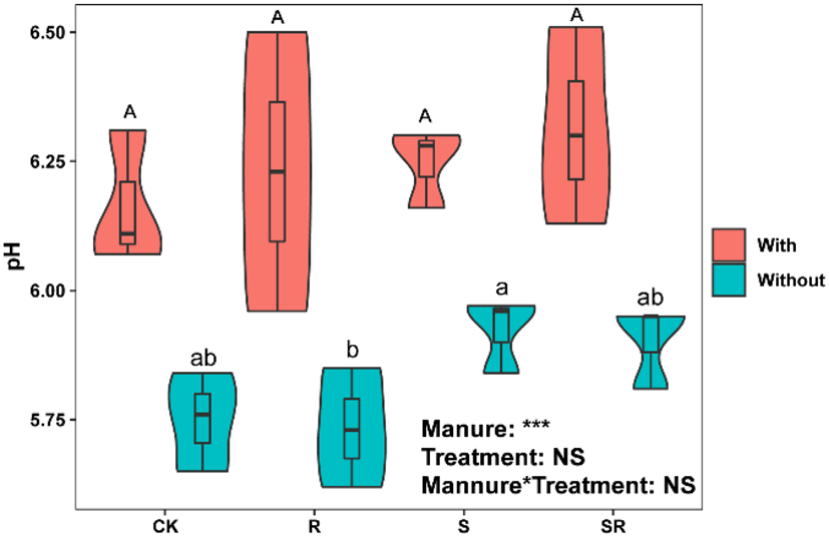
Effect of EOM on soil pH, and the plots were labeled without pig manure (without), with pig manure (with). The data were expressed by mean ± standard error (n = 3). Pig manure × Treatment: interaction between crop residues without and with pig manure. The different lowercase and uppercase letters denote the significant difference among different treatments without and with pig manure addition at 0.05 level, respectively. The two-way ANOVA results were indicated by NS non-significant, *p < 0.05, **p < 0.01, ***p < 0.001.

Two-way ANOVA showed that crop residues and pig manure addition, and their interactions significantly influenced the contents of MBN and MBP (*p* < 0.05, Figs. 1e and f). TC and MBN contents significantly increased in both S and SR treatments compared with CK regardless of whether pig manure was added (Figs. 1a and e). Pig manure addition increased TC and MBN contents in all the treatments compared with the corresponding treatments without pig mnaure, except for R treatment (Figs. 1a and e). Compared with CK, MBC content significantly increased and reduced in both S treatment and R treatment with pig manure, respectively (Fig. 1d), while significantly increased in SR treatment without pig manure (Fig. 1d). There were no significant differences in TP, DOC, AP, NH_4_^+^–N and NO_3_^−^–N contents, and pH value among crop residues treatments compared with CK regardless of whether pig manure was added, except for DOC and NO_3_^−^–N contents in SR treatment without pig manure (Figs. 1c, 2a–d and 3, and Figs. 2a and d). However, pig manure addition increased contents of TP, DOC, AP and NH_4_^+^–N, and pH value in all the treatments compared with the corresponding treatments without pig manure (Figs. 1c, 2a–c and 3). Compared with CK, TP and MBP contents were not significantly influenced by crop residues regardless of whether pig manure was added (Figs. 1c and f), while TN content significantly increased in crop residue treatment without pig manure, and MBP content was significantly higher in S treatment with pig manure, respectively (Figs. 1b and f).

### Soil enzyme activities and microbial community composition under EOM additions

**Figure 4:**
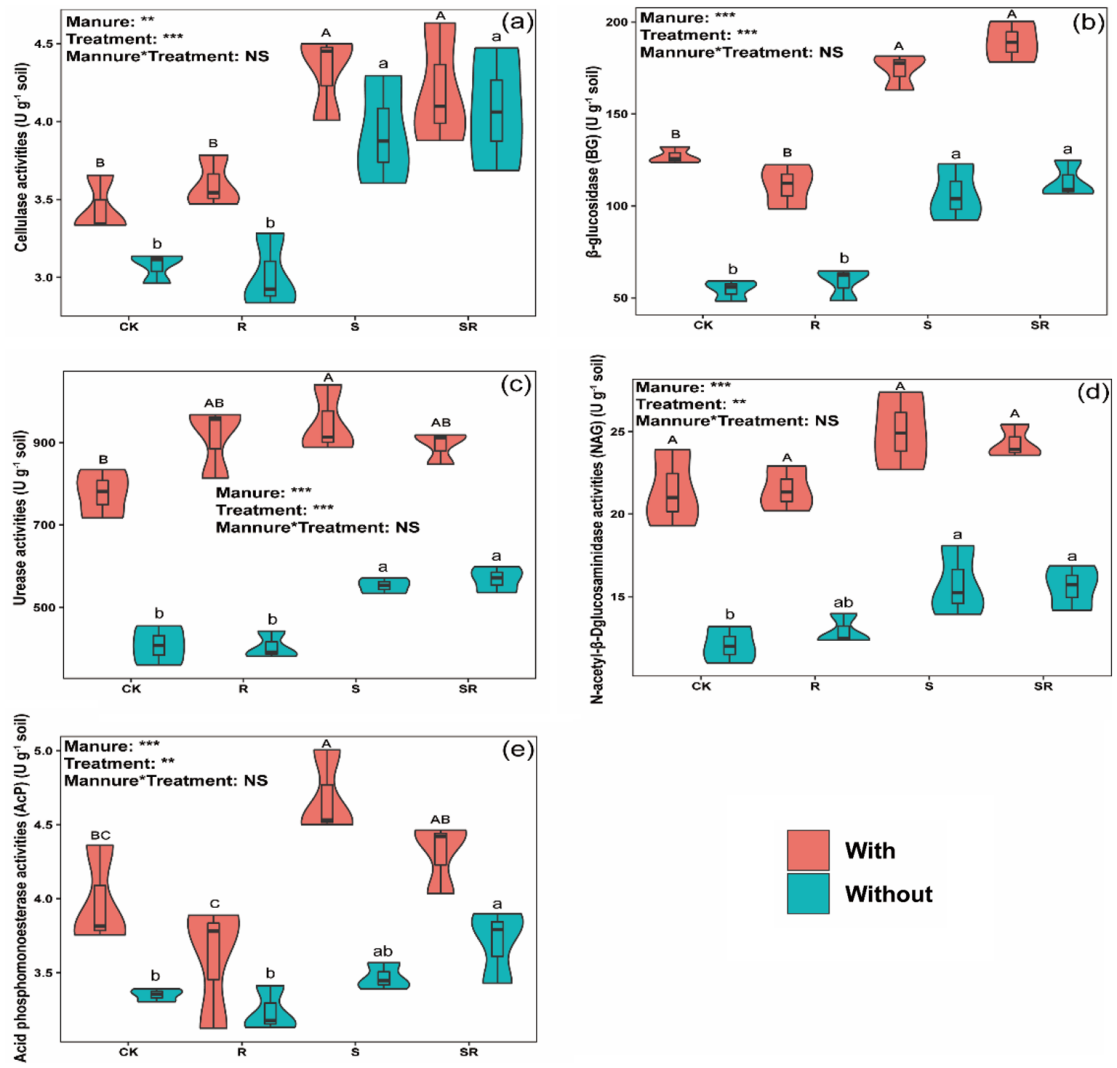
Effect of EOM on soil enzymes activities, and the plots were labeled without pig manure (without), with pig manure (with). The data were expressed by mean ± standard error (n = 3). Pig manure × Treatment: interaction between crop residues without and with pig manure. The different lowercase and uppercase letters denote the significant difference among different treatments without and with pig manure addition at 0.05 level, respectively. The two-way ANOVA results were indicated by NS non-significant, *p < 0.05, **p < 0.01, ***p < 0.001.

Compared with CK, both S and SR significantly increased cellulase and BG activities (Figs. 4a and b) regardless of whether pig manure was added. Urease and AcP activities were significantly higher in the S treatment with pig manure than that in CK (Figs. 4c and e). Urease and NAG activities were significantly higher in S and SR treatment without pig manure than those in CK (Figs. 4c and d), while the AcP activity significantly increased in SR treatment without pig manure (Fig. 4e). Pig manure addition increased activities of BG, urease, NAG and AcP in all the treatments compared with the corresponding treatments without pig manure, except for AcP activity in R treatment (Figs. 4b–e).

**Figure 5:**
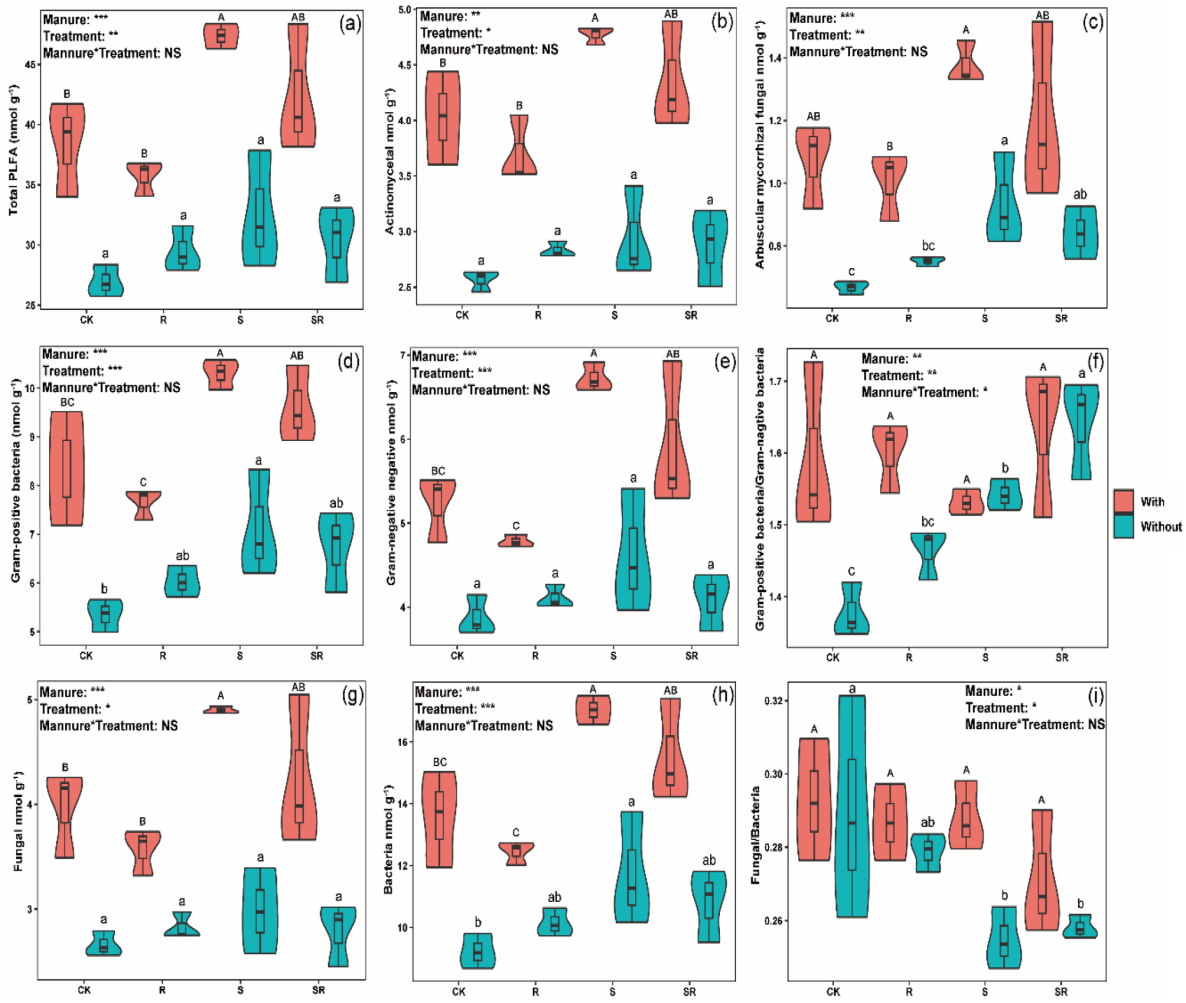
Effect of EOM on PLFA content of soil microbial communities, and the plots were labeled without pig manure (without), with pig manure (with). The data were expressed by mean ± standard error (n = 3). Pig manure × Treatment: interaction between crop residues without and with pig manure. The different lowercase and uppercase letters denote the significant difference among different treatments without and with pig manure addition at 0.05 level, respectively. The two-way ANOVA results were indicated by NS non-significant, *p < 0.05, **p < 0.01, ***p < 0.001.

Two-way ANOVA showed that crop residues and pig manure addition, and their interactions significantly influenced G+/G− ratio (*p* < 0.05, Fig. 5f). Compared with CK, the S treatment with pig manure significantly increased the contents of total PLFA, and actinomycetal, G+, G-, fungi and bacteria PLFAs (Figs. 5a, b,d, e, g, h), and the S treatment without pig manure significantly increased G+ and bacteria PLFAs contents (Figs. 5d and h), respectively, while S and SR treatment without pig manure significantly increased AMF PLFA content, G+/G- ratio, and significantly reduced F/B ratio, respectively (Figs. 5c, f and i). Pig manure addition increased total PLFA, and actinomycetal, AMF, G+, G−, fungi and bacteria PLFAs contents in all the treatments compared with the corresponding treatments without pig manure (Figs. 5a–e, g and h).

### Relationship among soil microbial communities, soil properties and enzyme activities

The results of Pearson correlation analysis (Table 2) showed that BG activity was significantly positively correlated with G+, AMF PLFAs contents and G+/G− ratio, while significantly negatively correlated with F/B ratio; cellulase activity was significantly positively correlated with G+/G− ratio, and negatively correlated with F/B ratio; urease and NAG activities were significantly positively correlated with AMF PLFA content and G+/G− ratio, and negatively correlated with F/B ratio under crop residues without pig manure addition, respectively. For crop residues with pig manure addition, BG activity was significantly positively correlated with G+, G−, actinomycetal and bacteria PLFAs contents; AcP activity was significantly positively correlated with G+, G− and bacteria PLFAs contents, respectively.

**Table 2:** Correlations between soil microbial communities and soil enzyme activities under EOM addition.

Treatment	Index	PLFA of soil microbial communities							
		G+	G−	Actinomycetal	Fungal	Bacterial	AMF	F/B	G+/G−
Without pig manure	Cellulase	ns	ns	ns	ns	ns	ns	− 0.63*	0.68*
	BG	0.66*	ns	ns	ns	ns	0.68*	− 0.74**	0.87**
	Urease	ns	ns	ns	ns	ns	0.61*	− 0.80**	0.75**
	NAG	ns	ns	ns	ns	ns	0.58*	− 0.72**	0.78**
	AcP	ns	ns	ns	ns	ns	ns	ns	ns
With pig manure	Cellulase	ns	ns	ns	ns	ns	ns	ns	ns
	BG	0.74**	0.68*	0.59*	ns	0.73**	ns	ns	ns
	Urease	Ns	ns	ns	ns	ns	ns	ns	ns
	NAG	ns	ns	ns	ns	ns	ns	ns	ns
	AcP	0.60*	0.59*	ns	ns	0.60*	ns	ns	ns

Significant values are in bold.

*G*+ Gram-positive bacteria, *G − *Gram-negative bacteria, *AMF* arbuscular mycorrhizal fungi, *F/B* the ratio of fungal to bacterial, *G*+*/G− *the ratio of Gram-positive bacteria to Gram-negative bacteria, *BG* β-1,4-glucosidase, *NAG N*-acetyl-β-Dglucosaminidase, *AcP* acid phosphomonoesterase.

*Significant correlation at 0.05 level (both sides), **significant correlation at 0.01 level (both sides), ns: no-significant.

**Figure 6:**
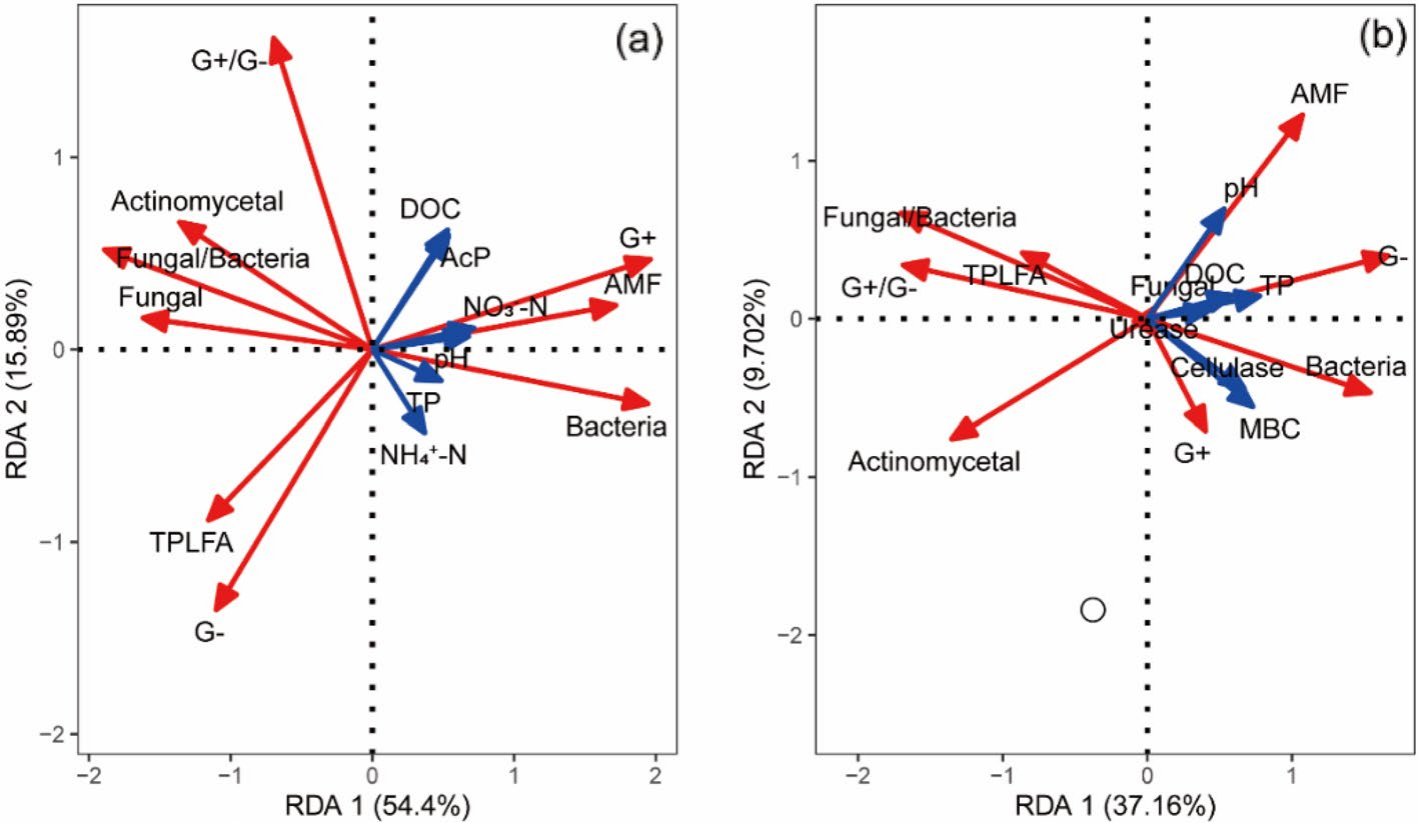
Redundancy analysis (RDA) between soil microbial communities and soil environmental parameters under EOM addition, and the plots were labeled without pig manure (**a**), and with pig manure (**b**). *NO*_*3*_^*−*^*–N* nitrate nitrogen; *NH*_*4*_^+^*–N* ammonia nitrogen, *DOC* dissolved organic carbon, *TP* total phosphorus, *MBC* microbial biomass carbon, *G*+ gram-positive bacterial, *G− *gram-negative bacteria, *AMF* arbuscular mycorrhizal fungi, *G*+*/G− *the ratio of gram-positive bacteria to gram-negative bacteria, *AcP* acid phosphomonoesterase.

The changes in soil microbial communities under crop residues with or without pig manure were analyzed by the redundancy analysis (RDA) (Fig. 6). RDA indicated that 37.16% and 9.70% of the variability in microbial community composition could be explained by the first and second principal components under crop residues with pig manure treatment, respectively (Fig. 6b and Table 3). Similarly, the changes in soil microbial community composition under crop residues without pig manure treatment was shown with the first and second principal components (RDA1 and RDA2) accounting for 54.40% and 15.89% of the variance, respectively (Fig. 6a and Table 3). This analysis also revealed that pH, the contents of NO_3_^−^–N, NH_4_^+^–N and DOC were significantly associated with soil microbial community composition under crop residues without pig manure addition (Fig. 6a and Table 3), whereas the correlation between explanatory variables and microbial community composition under crop residues with pig manure addition was not significant (Fig. 6b and Table 3).

**Table 3:** Statistic results of redundancy analysis (RDA) between soil microbial communities and explanatory environmental parameters under EOM addition.

Explanatory variables	Microbial composition without pig manure			Explanatory variables	Microbial composition with pig manure		
	Variance	F	*p-*value		Variance	F	*p*-value
pH	2.45	7.73	**	pH	1.38	1.80	0.188
NO_3_^−^–N	1.05	3.32	*	DOC	0.62	0.81	0.541
NH_4_^+^–N	1.55	4.89	*	TP	0.94	1.22	0.339
DOC	1.07	3.38	*	MBC	1.48	1.92	0.157
TP	0.60	1.91	0.165	Cellulase	0.35	0.45	0.797
AcP	0.69	2.19	0.122	Urease	0.41	0.53	0.728

*Significant correlation at 0.05 level; **significant correlation at 0.01 level.

*NO*_*3*_^*−*^*–N* nitrate nitrogen, *NH*_*4*_^+^*–N* ammonia nitrogen, *DOC* dissolved organic carbon, *TP* total phosphorus, *MBC* microbial biomass carbon, *MBN* microbial biomass nitrogen, *AcP* acid phosphomonoesterase.

*p < 0.05; **p < 0.01; ***p < 0.001.

## Discussion

### Effect of EOM on soil properties and microbial biomass

Returning crop residues to field can provide abundant essential nutrients such as carbon (C), nitrogen (N) and phosphorus (P), thereby enhancing soil fertility^36,37^. Our results indicated that both S and SR treatment significantly increased TC and MBN contents regardless of whether pig manure was added (Figs. 1a, e), and S treatment with pig manure and SR without pig manure significantly increased MBC content (Fig. 1d). These results suggest that crop straw can influence soil microbial biomass and activities by altering the supply of C and other nutrients. We also found that MBC significantly increased in S treatment and significantly reduced in R treatment with pig manure, while significantly increased in SR treatment without pig manure compared with CK, respectively (Figs. 1d). The above results might be attributed to the carbon to nitrogen ratio (C/N) and the biochemical composition of crop residues. Previous studies reported that crop residues with high C/N of rice straw might cause soil microorganisms to compete with the crops for N, while high C/N might also reduce the accumulation of available N through microbial immobilization processes^38,39^. Conversely, the root with a low C/N (R treatment) might enhance the mineralization, thereby decreasing MBC and MBN contents (Figs. 1d, e). Our study found the interaction between crop residues and pig manure significantly influenced MBN and MBP contents (Figs. 1e, f), suggesting that pig manure addition might alter soil biological properties and regulated the keystone microbial groups involved in crop residues decomposition^40^.

Moreover, the DOC content increased mainly due to the release of soluble organic matter and some nutrients (e.g., N, P) from the C inputs or crop residues decomposition, which stimulated microbial activities and growth^41^. In our study, pig manure addition increased TP, DOC, AP and NH_4_^+^–N contents, and pH value in all the treatments (Figs. 1c, 2a–c, 3), suggesting that pig manure addition improves soil fertility by increasing the organic matter, nutrients availability (e.g., available N and P), and elevating soil pH. In addition, pig manure is enriched with high organic matter, N and P nutrient contents^10^, and could further increase DOC and TP contents to higher levels after applied to the soil.

### Effect of EOM on soil enzyme activities

Soil enzymes are considered as indicators of soil fertility, and are involved in SOM transformation and nutrients turnover^42^. Changes in soil fertility depend on the changes in soil enzymes activities, and some hydrolytic enzymes such as cellulase, BG, urease, NAG and AcP are associated with soil C, N and P cycling^12^. Compared with CK, both S and SR significantly increased cellulase and BG activities (Figs. 4a, b) regardless of whether pig manure was added, and urease activities significantly increased in S with and without pig manure, and NAG activities were significantly higher in S and SR treatment without pig manure (Figs. 4c, d), respectively. This may have occurred because the microbial population and microbial biomass C or N were increased by EOM addition, which provided organic matter that is used as a substrate for soil enzymes^43,44^. It was recently reported that the straw with easily decomposable matter could improve the availability of P in soils by providing energy materials for microbial activities and abundant substrates for enzymatic processes^45^. Our study revealed that the AcP activity significantly increased in the S treatment with pig manure and SR treatment without pig manure than that in CK, respectively (Fig. 4e). Pig manure addition increased BG, urease, NAG and AcP activities in all the treatments, except for AcP activity in R treatment (Figs. 4b–e). This is probably because pig manure addition affected the unstable pool of soil organic nutrients, thereby influencing enzyme activities^46^. However, soil enzymes activities are regulated by soil C and N levels, and the lower decomposition rates of roots might limit the substrate use efficiency for soil enzymes in R treatment. Therefore, crop residues and pig manure addition might affect soil enzyme activities by influencing the quantity and quality of C inputs.

### Effect of EOM on soil microbial community composition

Compared with CK, the presence of rice straw increased the total PLFA, and actinomycetal, G+, G−, fungi, bacteria and AMF PLFAs contents, and G+/G− ratio, and reduced F/B ratio, respectively. These phenomena were attributed to the preference of different microorganisms for C substrates. Specifically, total PLFA significantly increased in the S treatment with pig manure compared with CK (Fig. 5a), this is probably because crop residues (e.g., straw, roots) and pig manure could provide energy and substrates for microbial metabolisms and growth^12,46^. Previous studies have reported that actinomycetal are efficient decomposers of C-poor compounds, so they exhibit the highest activity when soil is C-poor due to N limitation^47^. We observed a significant increase in actinomycetal in S treatment with pig manure compared with CK (Fig. 5b), this is probably because the straw and pig manure with a higher C/N ratio might cause N limitation by soil microorganism competing with crops for N. Our results indicated that AMF PLFA content significantly increased in S and SR treatment without pig manure (Fig. 5c), probably because AMF could also recycle and distribute nutrients from older mycelia to newly grown mycelia^48^, so they are less affected by active nutrients. Our results also showed that the interaction between crop residues and pig manure significantly increased the G+/G− ratio in S and SR treatment without pig manure (Fig. 5f), suggesting that the presence of rice straw increased the relative abundance of G+ bacteria more than that of G− bacteria (Figs. 5d, e). This might be because G+ bacteria are more stress-tolerant than G− bacteria, and G+ bacteria prefer to use recalcitrant substrates, whereas G− bacteria prefer to use relative labile residue C^49,50^.

Bacteria and fungi are the two main decomposers, of which bacteria are better adapted to the metabolism of easily decomposed organic matter, and fungi have an advantage in soil with high contents of recalcitrant organic matter (e.g., cellulose and lignin)^51,52^. In our study, bacteria and fungi PLFAs contents significantly increased in S treatment with pig manure while bacteria PLFA content significantly increased only in S treatment without pig manure (Figs. 5g, h), indicating that pig manure could provide abundant available nutrients for bacteria and fungi, whereas straw was readily degradable to release nutrients that can meet the metabolic requirement of bacteria. The F/B ratio is capable to characterize the most active microbial community for degrading crop residues^53^. In addition, our results found that the S and SR treatment significantly decreased the F/B ratio under no pig manure (Fig. 5i), and a significantly negative correlation existed between the F/B ratio and the C, N acquisition enzymes (e.g., cellulase, BG, urease and NAG) under straw without pig manure (Table 1). These results indicate that enzymatic hydrolysis released available nutrients for microbial growth and metabolism, resulting in a higher level of bacterial community than the fungal community.

### Potential relationships between soil microbial communities and soil enzymes activities and soil properties

In our study, crop residues with or without pig manure treatment significantly affected the content of total PLFAs and altered microbial community composition (Figs. 5, 6). These changes might attribute to the organic materials containing labile and recalcitrant organic C^54^, which could promote microbial metabolism and growth. In addition, the increases in microbial biomass (C, N and P) and available nutrients (C, N and P) contents were observed under crop residues combined with pig manure addition (Figs. 1, 2), which could provide energy and substrates for microbial activity. This result was further confirmed by RDA analysis, which revealed that the relationships between microbial community composition and soil properties reached 46.86% and 70.39% of the variability under crop residues with and without pig manure treatment, respectively (Fig. 6). Specifically, the changes in the microbial community composition were dependent upon pH, NO_3_^−^–N, NH_4_^+^–N and DOC contents under crop residues without pig manure treatment, whereas the correlation between explanatory variables and microbial community composition under crop residues with pig manure addition was not significant (Fig. 6b; Table 3), indicating that microbial community was not limited by the available nutrients under pig manure addition. Moreover, we also found that the higher microbial biomass (C, N and P) and available nutrients (C, N and P) contents were observed in pig manure treatment compared with the treatments without pig manure (Fig. 1, 2). This was probably because the relatively abundant energy and substrates were enough to maintain the microbial metabolism and activity.

Generally, nutrient turnover was related to microbial community composition and extracellular enzymes activities, and the correlation between microbial community composition and extracellular enzymes was significant^46,55,56^. Moreover, Tasoff et al.^57^ have predicted the increase in enzyme production when simple nutrients are scarce and complex nutrients are abundant by using economics of microbial metabolism. In our study, the F/B ratio was significantly negatively correlated with the C, N acquisition enzymes (i.e., cellulase, BG, urease and NAG) activities under straw treatment without pig manure (Table 1), indicating that changes in microbial community composition might depend on soil condition such as C and N availability^58^, as well as competition for available nutrients with C, N acquisition enzymes. However, the G+/G− ratio was significantly positively correlated with the C, N acquisition enzymes (i.e., cellulase, BG, urease and NAG) under straw without pig manure (Table 1), suggesting that straw addition shifted the microbial community towards a more G+ bacteria-dominated stage^59^. Previous study also found that G+ bacteria are capable of secreting enzymes to decompose recalcitrant C, but that they require higher N levels^60^. Furthermore, organic matter mineralization is necessary to provide energy and substrates for G+ with the depletion of available nutrients, whereas the recalcitrant compounds are more readily degraded by extracellular enzymes^61,62^. Fungi could degrade cellulose or recalcitrant components (e.g., recalcitrant C, organic N and P polymers) by secreting soil extracellular enzymes^51,63^. In addition, mycorrhizal fungi could form symbiotic relationships with plant roots facing nutrients deficiency stress, thus releasing mineralized nutrients such as N and P^64,65^. In our study, AMF PLFA content was significantly positively with BG, urease and NAG activities under crop residues without pig manure (Table 1), indicating that the mineralization process requires mycorrhizal fungi to produce extracellular enzymes to decompose C and N compounds, and then facilitate microbial metabolisms and growth. Bacteria play an important role in organic matter decomposition, where G+ bacteria are more suitable for soil with low substrate availability^66^. The early catabolism of G+ bacteria is dependent on more labile substrates, and the labile substrates require BG involvement in the crop residues mineralization^67^. In addition, we also observed the positive correlation between G+ bacteria PLFA content and BG activity (Table 1). These results indicated that G+ bacteria play more important roles in the mineralization process of crop residues treatment without no pig manure addition.

The relationship between microbial community composition and enzyme activities exhibited difference under crop residues treatments with pig manure addition. That’s probably because pig manure can increase soil fertility by releasing large amounts of organic matter, N and P that are beneficial to microorganisms^68–70^. Moreover, DOC, as an unstable C fraction, is considered to be the dominant source of substrates and energy for microorganisms^71^. We found that pig manure significantly increased DOC content (Fig. 2a) and BG activity (Fig. 4b). The higher BG activity further promoted the mineralization of crop residues with increasing energy and substrates. Although crop residues decomposition is a complex process, pig manure provides durable energy and nutrients for a good microbial community structure. Our finding indicated that the BG activity was significantly positively with the PLFAs contents of G+ bacteria, G− bacteria, actinomycetal and bacteria under crop residues with pig manure (Table 2), this might be a direct effect of pig manure. It has been confirmed that pig manure addition can increase the total P and soluble P contents in soil, and provide a good nutrient source to supplement soil P for crops growth^70^. Moreover, the increase in moderately unstable and stable P is possibly due to the adsorption or precipitation process, some of which may also come from pig manure and may not be readily absorbed by plants^72^. In our study, the PLFAs contents of bacteria (including G+ bacteria and G− bacteria) were significantly and positively correlated with AcP activity (Table 2), suggesting that pig manure could stimulate the activity of AcP by providing good P sources for microorganisms, as well as C, N sources. Thus, pig manure not only provides rich nutrients (C, N and P) for microorganisms but also induces the higher of enzyme activities to maintain microbial activities and diversity.

## Conclusions

This study showed that exogenous organic matter addition increased microbial biomass and enzymes activities. Relative to the below-ground roots (R) treatment, the high carbon to nitrogen ratio (C/N) of straw could reduce the accumulation of available nutrients through microbial immobilization process. Moreover, exogenous organic matter addition could provide substrates for soil enzymes by influencing the quantity and quality of carbon inputs. Compared with CK, the presence of straw increased the contents of total PLFA, and actinomycetal, gram-positive bacteria, gram-negative bacteria, fungi, bacteria and AMF PLFAs, gram-positive bacteria to gram-negative bacteria ratio (G+/G−), and reduced fungi to bacteria ratio (F/B), respectively, indicating the preference of different microorganisms for C substrates. Redundance analysis confirmed that pH, nitrate nitrogen (NO_3_^−^–N), ammonium nitrogen (NH_4_^+^–N) and dissolve organic carbon (DOC) contents were dominant factors in regulating the changes of soil microbial community under crop residue treatment without the pig manure, whereas the nutrients were relatively abundant to maintain microbial metabolism and activity under crop residues with the pig manure treatment. Overall, the combination of above-ground straw and pig manure was more beneficial to improve soil carbon and nitrogen availability, increase enzyme activities and facilitate microbial biomass and activity compared with no pig manure. Our findings suggest that the combination of above-ground straw and pig manure is a better alternative for improving the functions of soil ecosystem, especially for no-tillage with straw management system.

## Data Availability

The dataset generated in the course of the current study can be obtained from the corresponding author upon reasonable request.
